# Colossal dielectric permittivity, reduced loss tangent and the microstructure of Ca_1−*x*_Cd_*x*_Cu_3_Ti_4_O_12−2*y*_F_2*y*_ ceramics

**DOI:** 10.1039/d1ra02707g

**Published:** 2021-05-04

**Authors:** Jakkree Boonlakhorn, Jirata Prachamon, Jedsada Manyam, Sriprajak Krongsuk, Prasit Thongbai, Pornjuk Srepusharawoot

**Affiliations:** Giant Dielectric and Computational Design Research Group (GD-CDR), Department of Physics, Faculty of Science, Khon Kaen University Khon Kaen 40002 Thailand spornj@kku.ac.th; National Nanotechnology Center (NANOTEC), National Science and Technology Development Agency (NSTDA) Pathum Thani 12120 Thailand; Institute of Nanomaterials Research and Innovation for Energy (IN-RIE), NANOTEC-KKU RNN on Nanomaterials Research and Innovation for Energy, Khon Kaen University Khon Kaen 40002 Thailand

## Abstract

Ca_1−*x*_Cd_*x*_Cu_3_Ti_4_O_12−2*y*_F_2*y*_ (*x* = *y* = 0, 0.10, and 0.15) ceramics were successfully prepared *via* a conventional solid-state reaction (SSR) method. A single-phase CaCu_3_Ti_4_O_12_ with a unit cell ∼7.393 Å was detected in all of the studied ceramic samples. The grain sizes of sintered Ca_1−*x*_Cd_*x*_Cu_3_Ti_4_O_12−2*y*_F_2*y*_ ceramics were significantly enlarged with increasing dopant levels. Liquid-phase sintering mechanisms could be well matched to explain the enlarged grain size in the doped ceramics. Interestingly, preserved high dielectric permittivities, ∼36 279–38 947, and significantly reduced loss tangents, ∼0.024–0.033, were achieved in CdF_2_ codoped CCTO ceramics. Density functional theory results disclosed that the Cu site is the most preferable location for the Cd dopant. Moreover, F atoms preferentially remained close to the Cd atoms in this structure. An enhanced grain boundary response might be a primary cause of the improved dielectric properties in Ca_1−*x*_Cd_*x*_Cu_3_Ti_4_O_12−2*y*_F_2*y*_ ceramics. The internal barrier layer capacitor model could well describe the colossal dielectric response of all studied sintered ceramics.

## Abstract 2

CdF_2_ defect clusters result in enhancement of dielectric properties of the Ca_1−*x*_Cd_*x*_Cu_3_Ti_4_O_12−2*y*_F_2*y*_ ceramics.

## Graphical abstract

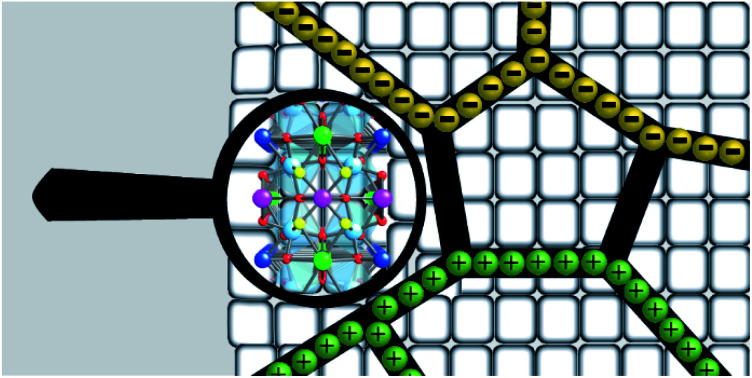

## 1. Introduction

Over the past decade, the survey of colossal dielectric permittivity (*ε*′ ∼ 10^3^ to 10^5^) materials has been one of the most fascinating topics in the fields of energy-storage and electronic applications.^1–24^ In advanced ceramics, essential raw materials for producing electronic components have been widely researched in this important field. Focusing on capacitor applications, the colossal dielectric properties of polycrystalline ceramics, such as the codoped TiO_2_,^1,2,17,20–22^ codoped SnO_2_,^3^ and ACu_3_Ti_4_O_12_ (A = Ca,^4–9,24^ Cd,^10–12^ Sm_2/3_,^13^ Y_2/3_,^14^ Na_1/2_Y_1/2_,^15^ Na_1/2_Sm_1/2_,^25^ Na_1/3_Cd_1/3_Y_1/3_ ^16^ ceramics have been developed and reported in the literature. Preserving high *ε*′ values in these ceramics is the primary objective, followed by reducing loss tangents (tan *δ*) and enhancing temperature/frequency stability. Reports related to CaCu_3_Ti_4_O_12_ (CCTO) based ceramics have been continuously published.^4–9,26–29^ Due to its high *ε*′, numerous researchers have proposed an internal barrier layer capacitor (IBLC) structure, consisting of semiconducting grains and insulating grain boundaries (GBs). This was the first model used to explain the colossal dielectric response in ACu_3_Ti_4_O_12_ ceramics.^9–13,16,25–27^ However, other factors, *e.g.*, sample-electrode contact,^27^ charge defects or ionic defects,^28^ also have essential roles in the dielectric and electrical responses of ACu_3_Ti_4_O_12_ ceramics that cannot be neglected. High *ε*′ and non-ohmic properties measured in CCTO ceramics mainly originate from the insulating GB layers in IBLC structures.^30^

The most severe problem of CCTO for capacitor applications is its rather high tan *δ*. Currently, modification of the preparation process and substitution of ions into the CCTO structure are frequently used to improve its dielectric and non-ohmic properties.^4–8,26–29^ Additionally, modification of the A site of ACu_3_Ti_4_O_12_ ceramics has also been a popular approach.^10–12,14–16,25^ As previously reported by Yang *et al.*, showed that doping Li^+^, Zn^2+^, Mg^2+^, and Zr^4+^ into the CdCu_3_Ti_4_O_12_ structure can result in high *ε*′ values of ∼1.0–5.0 × 10^4^ with low tan *δ* values of ∼0.03–0.10.^10–12,31^ Similarly, addition of a Cd^2+^ ion into the A sites of Na_1/3_A_1/3_Bi_1/3_Cu_3_Ti_4_O_12_ and Na_1/3_A_1/3_Y_1/3_Cu_3_Ti_4_O_12_ can improve the dielectric properties of these two ceramics as well.^16,32^ Thus, it is reasonable to suggest that Cd^2+^ ions can have a crucial role in developing high *ε*′ and low tan *δ* values. In our previous work with (Sr^2+^, Ge^4+^),^27^ (Li^+^, F^−^),^8^ and (Sr^2+^, F^−^)^7^ codoped CCTO ceramics, it was found that the dielectric response is greatly enhanced in these ceramics as well. There are few reports related to Cd^2+^ doped CCTO ceramics.^33,34^ Additionally, experimental work and intensive discussions have not resulted in a clear understanding of these materials. Cd^2+^ and F^−^ codoped CCTO ceramics are the focus of the current study. The roles of Cd^2+^ and F^−^ in CCTO are systematically investigated. For high concentrations of Cd^2+^ ions doped in the CCTO, various reports showed that Cd^2+^ ions replace Ca^2+^ ions entirely.^12,31^ However, at low concentrations, Cd^2+^ may replace ions at other sites, such as those of Cu^2+^. Due to it charge state, Cd^2+^ can occupy ion sites with coordination numbers (CNs) of either 4 or 12,^35^ which are host sites of either Cu^2+^ or Ca^2+^, respectively. Therefore, a computational method, *e.g.*, density functional theory (DFT) calculations, should be used to determine the most stable location of Cd in the CCTO host.

In this work, Ca_1−*x*_Cd_*x*_Cu_3_Ti_4_O_12−2*y*_F_2*y*_ ceramics with *x* = *y* = 0, 0.10, and 0.15 were fabricated using a conventional solid-state reaction (SSR) method. We systematically examined both the microstructure and crystal structure of all studied sintered ceramics. First-principles calculation was employed to scrutinize the most stable sites of Cd and F in the CCTO structure. Experimental and computational details along with their results are discussed in subsequent sections.

## 2. Experimental details

### 2.1. Ceramic preparation

We initially prepared the TiO_2_ (99.99% purity, Aldrich), CuO (99.0% purity, Sigma-Aldrich), CaCO_3_ (99.0% purity, Sigma-Aldrich), CdF_2_ (98.0% purity, Sigma-Aldrich), and C_2_H_5_OH (99.5% purity, RCI Labscan) which are used in a SSR method to synthesize Ca_1−*x*_Cd_*x*_Cu_3_Ti_4_O_12−2*y*_F_2*y*_ (*x* = *y* = 0, 0.10, 0.15) ceramics. Stoichiometric ratios of all-ceramic conditions were weighed and ball-milled in C_2_H_5_OH at 150 rpm for 24 h. We heated these mixed materials in an oven at 80 °C for 24 h to vaporize all liquid from the sample materials. The resulting dried precursors were ground. We calcined the mixed powders in air at 850 °C for 12 h and then ground until their particle sizes were very fine. The Ca_1−*x*_Cd_*x*_Cu_3_Ti_4_O_12−2*y*_F_2*y*_ powders were formed into pellet shaped samples. Pellets with diameters of ∼9.5 mm and thicknesses of ∼2 mm were sintered at 1050 °C for 6 h. Sintered Ca_1−*x*_Cd_*x*_Cu_3_Ti_4_O_12−2*y*_F_2*y*_ ceramics with *x* = *y* = 0, 0.10, 0.15 are referenced as CCTO, CdF10, and CdF15 ceramics, respectively.

### 2.2. Crystal structure measurements

The phase composition and crystal structures of all Ca_1−*x*_Cd_*x*_Cu_3_Ti_4_O_12−2*y*_F_2*y*_ ceramics were examined using an X-ray diffractometer (XRD, EMPYREAN, PANalytical B.V.). In our XRD measurements, we used 2*θ* ranging from 20 to 80°. All XRD spectra were analyzed using the Rietveld refinement method employing X'Pert HighScore Plus software, Version 3.0e. The adjusted parameters and coefficients in the Rietveld method are global parameters (zero shift and background parameters), scale factor, unit cell, atomic coordinates, and profile parameters. A desktop scanning electron microscope (MiniSEM, SEC, SNE-4500M) was used to identify microstructural changes. An energy-dispersive X-ray spectrometer (EDS), included in the MiniSEM, was used to determine the chemical composition of the grains and GBs of sintered ceramics.

### 2.3. Dielectric and electrical measurements

Prior dielectric and electrical measurements, the surfaces of the ceramic samples were polished. These polished surfaces were coated with a conductive silver paint (Heraeus, PCC11889). Then all ceramic samples were fired in air at 680 °C for 0.5 h. Dielectric and electrical properties of Ca_1−*x*_Cd_*x*_Cu_3_Ti_4_O_12−2*y*_F_2*y*_ ceramics were measured using a KEYSIGHT E4990A with an oscillation voltage (*V*_rms_) of 0.5 V. The dielectric properties were studied over the frequency and temperature ranges of 40 to 10^7^ Hz and −60 to 210 °C, respectively. The methods for calculation of dielectric and electrical parameters are given elsewhere.^15^

### 2.4. Computational details

The Vienna *Ab initio* Simulation Package (VASP)^36^ was used to calculate the total and formation energies of the Cd and F codoped CCTO ceramics. We used the Perdew–Burke–Ernzerhof (PBE)^37^ form of the exchange-correlation functional. For CCTO, the pseudopotentials of Ca, Cu, Ti, and O are given in ref. 27. According to the pseudopotential of Cd, 4d and 5s valence states were used. Additionally, the 2s and 2p states were chosen as valence states of F. A cutoff energy of 470 eV was successfully used to test for energy convergence. 1 × 3 × 5 *k*-points samplings in the reciprocal space were employed. In the present study, a 5 × 2 × 1 CCTO supercell containing a total of 400 atoms was used.

## 3. Results and discussion

**Fig. 1:**
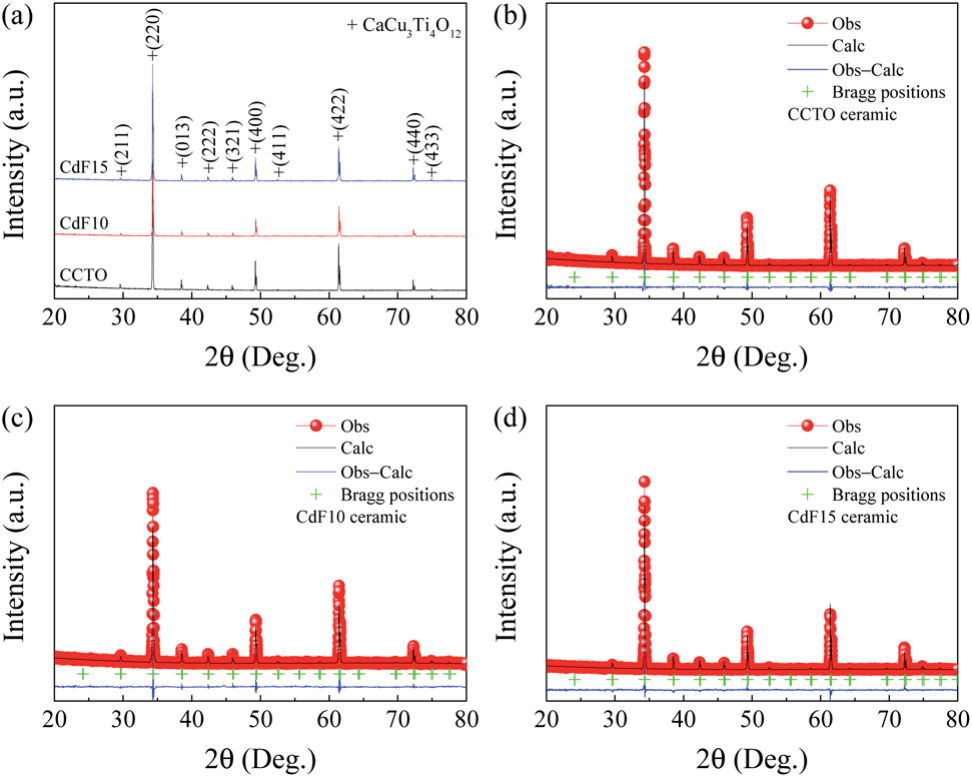
(a) XRD patterns of CCTO, CdF10, and CdF15 ceramics. (b–d) Rietveld profile fits of CCTO, CdF10, and CdF15 ceramics, respectively.

All structural parameters obtained from XRD measurements are summarized in Table 1. A crystalline examination of all Ca_1−*x*_Cd_*x*_Cu_3_Ti_4_O_12−2*y*_F_2*y*_ ceramics in the current study was completed using XRD. As revealed in Fig. 1(a), all XRD patterns are directly matched the standard structure of CCTO, referencing JCPDS code of 01-075-2188. All XRD patterns showed a body-centered cubic structure within an *Im*3̄ no. 204 space group^38^ and presented a single-phase of CCTO.

**Table 1:** Structural data obtained from the Rietveld refinement and mean grain size (*G*) for CCTO, CdF10 and CdF15 ceramics

Sample	CCTO	CdF10	CdF15
*a* (Å)	7.393(1)	7.393(8)	7.393(7)
*R*_exp_ (%)	6.756	8.294	7.585
*R*_p_ (%)	4.361	5.917	5.671
*R*_wt_ (%)	7.133	9.351	8.045
GOF	1.118	1.271	1.125
*G* (μm)	51.46 ± 13.76	114.20 ± 40.17	306.78 ± 109.10

No possible impurity phases were detected in any of the sintered ceramics. Rietveld refinement was used to calculate the vital parameters obtained from the XRD data of each ceramic sample. Fig. 1(b–d) show that the XRD data were well fitted by the Rietveld method. The *R*-factors, *i.e.*, the profile *R* factor (*R*_p_), weighted profile *R* factor (*R*_wp_) and expected *R* factor (*R*_exp_) of all Ca_1−*x*_Cd_*x*_Cu_3_Ti_4_O_12−2*y*_F_2*y*_ ceramics were below 9.5%. As a result, low goodness-of-fit (GOF) values ∼1.12–1.27 were attained. As summarized in Table 1, the lattice parameters (*a*) of sintered Ca_1−*x*_Cd_*x*_Cu_3_Ti_4_O_12−2*y*_F_2*y*_ ceramics were similar, approximately ∼7.393 Å. The *a* values obtained in this work are comparable to previously published data.^26–28,38^ Co-substitution of Cd^2+^ into Ca^2+^ sites and F^−^ into O^2−^ sites should cause a unit cell of CCTO to decrease significantly. This is because the ionic radius of Cd^2+^ (*r*_12_ = 1.31 Å) for the CN = 12 is smaller than that of Ca^2+^ (*r*_12_ = 1.35 Å).^35^ Concurrently, the ionic radius of F^−^ (*r*_4_ = 1.31 Å) is also smaller than that of O^2−^ (*r*_4_ = 1.38 Å).^35^ However, the average size of the unit cell is constant. Although replacement of Cd^2+^ ions into all Ca^2+^ sites (CdCu_3_Ti_4_O_12_) can correctly form a body-centered cubic structure within an *Im*3̄ space group,^10–12^ substitution of a small amount of Cd^2+^ into Ca^2+^ sites might yield different results. It is reasonable to suggest that a small concentration of Cd^2+^ could replace Cu^2+^ in the CCTO structure. This is because the ionic radius of Cd^2+^ (*r*_4_ = 0.78 Å) for the CN = 4 is larger than that of Cu^2+^ (*r*_4_ = 0.57 Å). Consequently, if the larger Cd^2+^ ions replace Cu^2+^ at smaller sites and F^−^ ions occupy O^2−^ sites, this may allow the size of the unit cell to remain constant.

**Fig. 2:**
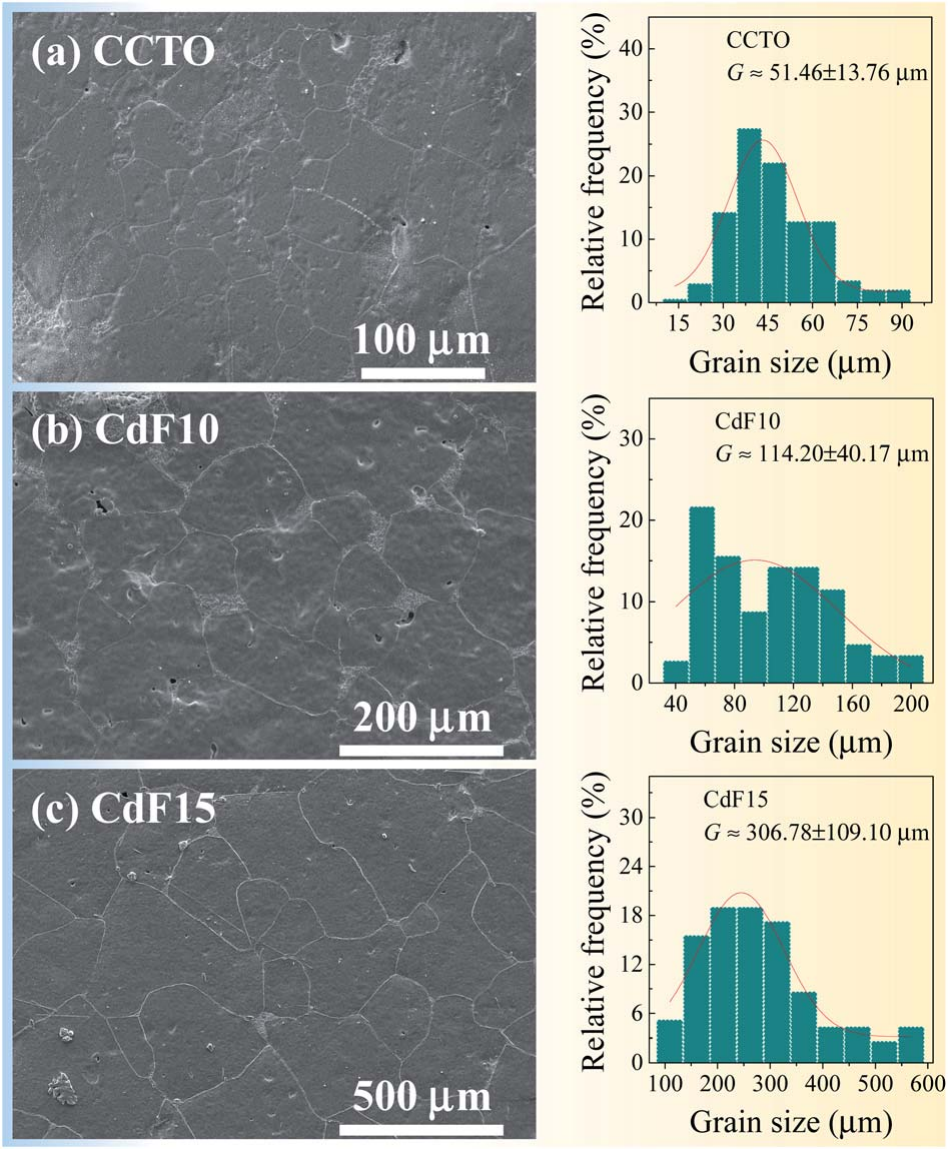
SEM images and size distributions of (a) CCTO, (b) CdF10, and (c) CdF15 ceramics.

SEM and ImageJ software were used to collect data and analyse the microstructure of ceramic samples. Microstructural imagery and size distributions of sintered Ca_1−*x*_Cd_*x*_Cu_3_Ti_4_O_12−2*y*_F_2*y*_ ceramics are shown in Fig. 2(a–c). Few pores can be seen in CCTO and CdF10 ceramics. Alternatively, highly dense ceramic material was observed in the CdF15 ceramic. The average grain size and size distributions of grains of ceramic samples were estimated using ImageJ software. To determine the average grain sizes of CCTO, CdF10, and CdF15 ceramics, we used ∼40 grains for CCTO and CdF10 samples and ∼30 grains for CdF15 ceramic in the current work. The average grain sizes of these ceramics are summarized in Table 1. They were found to be 51.46 ± 13.76, 114.20 ± 40.17, and 306.78 ± 109.10 μm, respectively.

The microstructural results reveal a significant increase in the grain sizes of the ceramic samples. A great increase in grain size due to doping was also reported in earlier published papers.^6–8,27^ This vastly increased grain size of the doped samples, compared to CCTO ceramic, might have been caused by liquid-phase sintering mechanisms. Generally, a liquid-phase can form at junctions between particles or GBs, resulting in a much increased diffusion rate. The liquid-phase might have originated from the direct melting of materials used in sample preparation or a reaction at a eutectic point between the raw metallic starting materials. As previously reported, a eutectic point between CuO–TiO_2_ could be formed at 950 °C.^39^ However, this reaction does have a great impact on the enormously expanded grain sizes of doped ceramics. Additionally, melting of CdF_2_ dopant during the sintering might not have been the cause of size expansion. According to previous research, the melting temperature of CdF_2_ is ∼1100 °C,^40^ much higher than the sintering temperature of CdF_2_ codoped CCTO ceramics (1050 °C). Hence, it is reasonable to propose that liquid-phase sintering mechanisms might be associated with replacing Cd^2+^ ions in the Cu^2+^ sites of CCTO structure during calcination at 850 °C for 12 h. Thus, a eutectic liquid of CuO–TiO_2_ is much more likely created due to surplus Cu ions, resulting in significantly enlarged grain sizes. This result is in agreement with our previously published work with (Sr^2+^, Ge^4+^) and (Ni^2+^, Ge^4+^) codoped CCTO.^9,27^

**Fig. 3:**
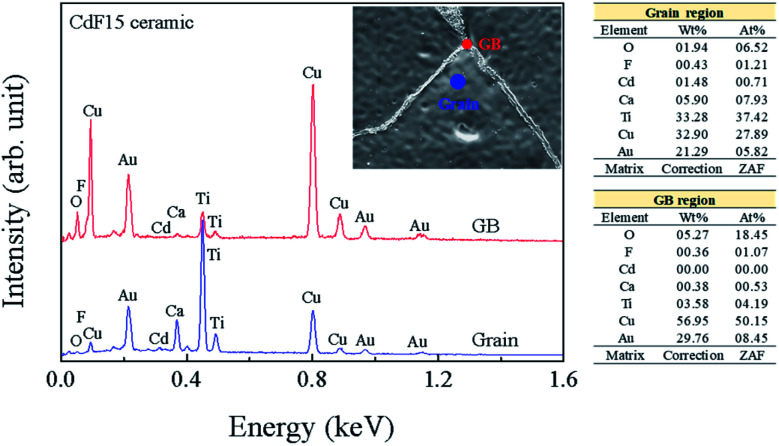
EDS spectrum and chemical compositions of the CdF15 ceramic at grain and GB regions.

The chemical constitutions of the GBs and grains of the CdF15 ceramic were examined using EDS. As shown in Fig. 3, Ca, Cu, Ti, and Cd contents were measured at the grain and GBs. At grain region, the Ti content was detected to be the highest followed by Cu, Ca, O and Cd, respectively. In addition, Cu contents, namely about 60% were mainly observed at GBs. It was found that the Cd^2+^ concentration detected inside the grains was much higher than that at the GBs, indicating the incorporation of Cd^2+^ into the CCTO lattice. An abundance of a Cu-rich phase was detected along the GB layers, as was seen in the EDS result. The presence of a Cu-rich phase directly relates to liquid-phase sintering mechanisms as a source of the increased grain sizes of the CdF_2_ codoped CCTO samples.^6–8,27^

**Fig. 4:**
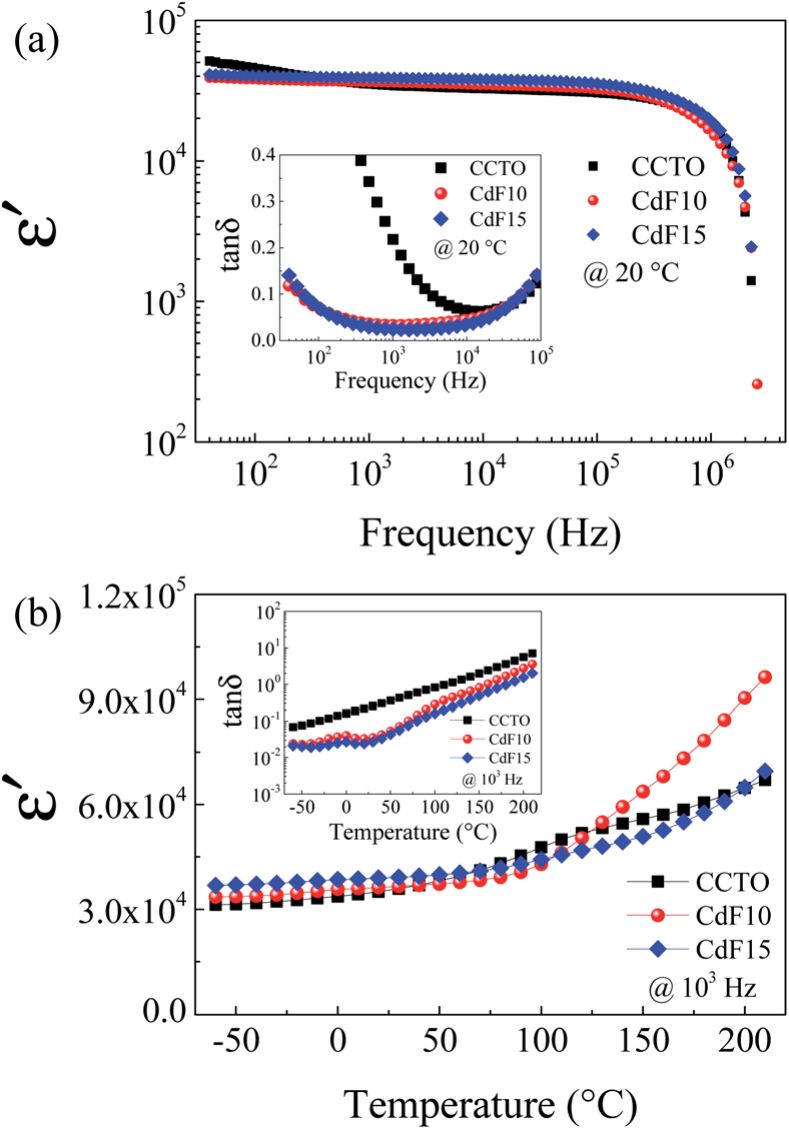
(a) Frequency dependence of *ε*′ at 20 °C of CCTO, CdF10, and CdF15 ceramics. Its inset displays frequency dependence of tan *δ*. (b) Temperature dependence of *ε*′ of the CCTO, CdF10, and CdF15 ceramics at 10^3^ Hz. The inset shows the temperature dependence of the tan *δ*.

Dielectric and electrical properties of CdF_2_ codoped CCTO ceramics were investigated under various conditions. The values of the parameters obtained are summarized in Table 2. Frequency dependence of *ε*′ at 20 °C of all sintered ceramic samples is shown in Fig. 4(a). The colossal dielectric response was detected over the entire frequency range of 40 to 10^6^ Hz. This result corresponds to its tan *δ* spectrum, as shown in the inset of Fig. 4(a).

**Table 2:** *ε*′ and tan *δ* at 1 kHz and 20 °C, resistances of grains (*R*_g_) at 20 °C and GBs (*R*_gb_) at 100 °C, activation energies of grains (*E*_g_) and GBs (*E*_gb_) of CCTO, CdF10 and CdF15 ceramics

Sample	*ε*′	tan *δ*	*R*_g_ (Ω cm)	*R*_gb_ (Ω cm)	*E*_g_ (eV)	*E*_gb_ (eV)
CCTO	35 047	0.218	50	5.52 × 10^4^	0.078	0.406
CdF10	36 279	0.033	56	2.86 × 10^5^	0.105	0.592
CdF15	38 947	0.024	47	4.32 × 10^5^	0.102	0.521

According to Li *et al.*,^41^ dielectric relaxation observed in the low-frequency range indicates the influence of DC conduction. In general, DC conduction in dielectrics can occur during the polarization process due to the long-range migration of charge carriers. It is notable that the frequency-independence of *ε*′ was achieved by doping with CdF_2_. The tan *δ* independence of frequency was observed in CdF10 and CdF15 ceramics over the frequency range of 40 to 10^5^ Hz. The *ε*′ value of CdF10 and CdF15 ceramics slightly increased compared to CCTO ceramic. This small increase in *ε*′ is closely associated with an increase in grain size. Therefore, the dielectric response of CdF_2_ codoped CCTO ceramics can be explained with an IBLC model.^6,26,27^ At 1 kHz and 20 °C, the *ε*′ values of CCTO, CdF10, and CdF15 were 35 047, 36 279, and 38 947, respectively. In the current work, not only was the *ε*′ value highly conserved, but the tan *δ* of CdF_2_ codoped CCTO ceramics was also reduced, as revealed in the inset of Fig. 4(a). tan *δ* values of CdF10 and CdF15 ceramics were respectively reduced to 0.033 and 0.024, compared to a value of 0.218 for the CCTO ceramic. The decrease in tan *δ* may have been induced by an enhanced GB response, as discussed in the next section. According to Maxwell–Wagner polarization, the *ε*′ value is reliant upon the GB capacitance (*C*_gb_).^8^ Although the grain sizes of the CdF10 and CdF15 ceramics were much larger than that of the CCTO sample, their *ε*′ values were only increased by ∼3.5–11.1%. Hence, the *ε*′ value of Ca_1−*x*_Cd_*x*_Cu_3_Ti_4_O_12−2*y*_F_2*y*_ ceramics might depend on both grain size and a change in *C*_gb_ due to CdF_2_ doping.^8^ Temperature dependences of *ε*′ and tan *δ* at 10^3^ Hz are shown in Fig. 4(b) and its inset, respectively. The *ε*′ of the CdF10 and CdF15 ceramics was found to be more stable than that of the CCTO ceramic in the temperature range below 120 °C. It is similar to that the influence of metastable-insulating layer at GB regions only affects the dielectric response of these codoped ceramics in the temperature range of −60 to 120 °C. Besides, the inset of Fig. 4(b) reveals that the tan *δ* of the CdF10 and CdF15 ceramics were much smaller than that of the CCTO sample through the temperature range of 60 to 120 °C. This dielectric result is likely to specifies the influence of co-substitution on improved dielectric response of CCTO ceramic. One possible origin of improved dielectric properties in the codoped CCTO ceramics might be the metastable-insulating phase along GB regions.^9,27,42^

**Fig. 5:**
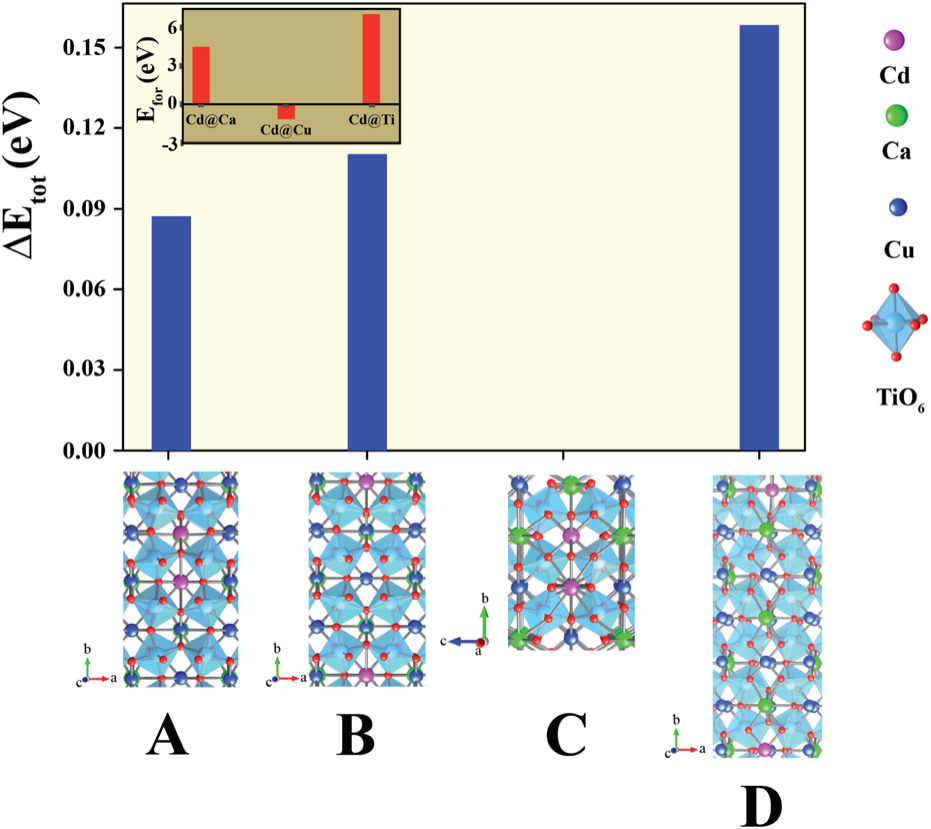
Total energy of four different structures, structures A–D of Ca_20_Cu_58_Cd_2_Ti_80_O_240_. Its inset represents the formation energy of Cd substituted at Ca, Cu and Ti sites in the 5 × 2 × 1 CCTO supercell.

As shown in Fig. 4(a), we found rather interesting results for the case of CdF10. Specifically, the *ε*′ value is very high and rather constant over a wide frequency range. Also, the tan *δ* of the CdF10 sample is indeed low in comparison to the CCTO ceramics. Based on these results, CdF10 was the primary focus. The stability of the CdF10 structure was determined by means of density functional theory calculations. Initially, we needed to test the location stability of a Cd atom in a CCTO host. This led to calculation of the formation energies when a Cd atom is substituted into Ca, Cu and Ti sites, as displayed in the inset of Fig. 5. Our results show that the formation energy of Cd@Cu is the lowest followed in order by those of Cd@Ca and Cd@Ti. In other words, Cd atoms are preferentially substituted into Cu sites. Consequently, Cu atoms move to the grain boundaries after Cd substitution. This results in liquid phase sintering at the grain boundaries. This finding is in excellent agreement with our experimental results as displayed in Fig. 3. Next, we needed to determine the most stable structure when two Cd atoms are replaced at Cu sites. In the present work, the total energy of four different configurations for the cases of two Cd atoms doped into the CCTO structure, namely structures A–D in Fig. 5, was determined.

**Fig. 6:**
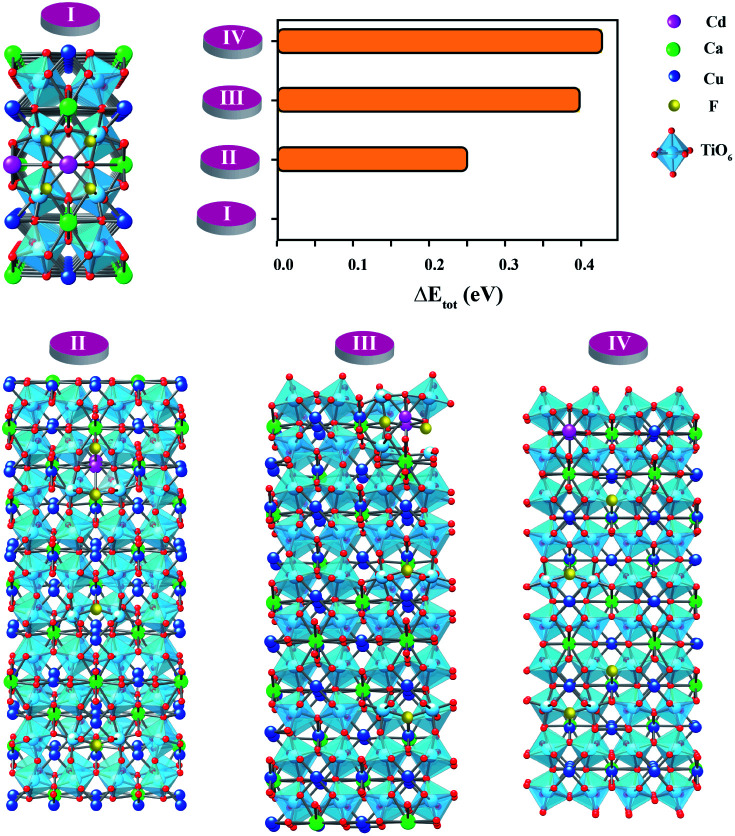
Total energy of various positions of F atoms in a CaCu_2.9_Cd_0.1_Cu_3_Ti_4_O_11.8_F_0.2_ or CdF10 host.

Based on our DFT results presented in Fig. 5, structure C presents the lowest total energy. As a result, structure C is the most stable structure for the Ca_20_Cd_2_Cu_58_Ti_80_O_240_ host. As clearly presented in Fig. 5, Cd atoms preferentially remain near each other. For the CdF10 or Ca_20_Cd_2_Cu_58_Ti_80_O_236_F_4_ structure, O was replaced by F atoms at four sites in the Ca_20_Cd_2_Cu_58_Ti_80_O_240_ and then the total energy of these structures was evaluated. In the current work, four different positions of F atoms in the Ca_20_Cd_2_Cu_58_Ti_80_O_236_F_4_ structure were considered, *i.e.*, structures I–IV in Fig. 6. Our DFT results revealed that total energy of structure I is the lowest followed by structures II, III and IV. In structure I of Fig. 6, four F atoms in the CdF10 are likely in close proximity. Consequently, the stable structure of CdF10 or Ca_20_Cd_2_Cu_58_Ti_80_O_236_F_4_ is structure I of Fig. 6.

**Fig. 7:**
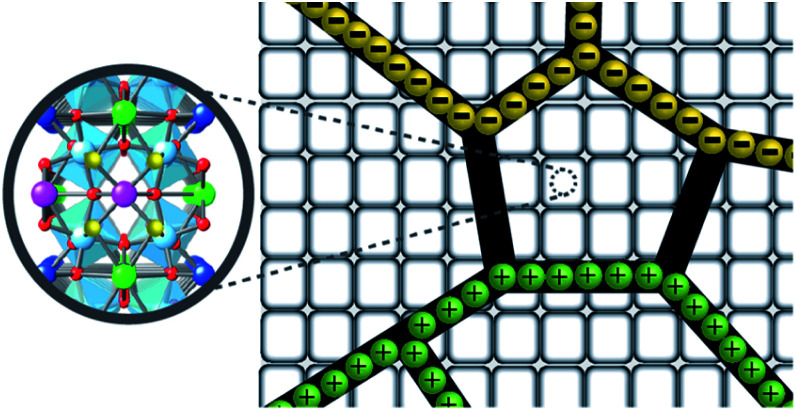
Insulating CdF_2_ cluster inside the grain of Cd and F codoped CCTO ceramics.

In the experimental and DFT results, it was found that co-substitution of Cd^2+^ and F^−^ can enlarge grain sizes and enhance dielectric properties. Although the grain sizes of CdF10 and CdF15 ceramics were greatly increased in comparison with the CCTO sample, the *ε*′ values of these two samples are only ∼3.5–11.1% larger. In general, the *ε*′ value of CCTO should increase with its grain size and more than double.^9,27,42^ However, this case is different. In structure I of Fig. 6, it is reasonable to suggest that the strong bonding of Cd^2+^–F^−^ atoms can form a small insulating barrier inside the grain and/or near GB regions. This insulating barrier is graphically presented in Fig. 7. As a result, this small insulating cluster might block or limit the zone for the migration of charge carriers during the dielectric polarization process, causing a decreased *ε*′ value. According to Maxwell–Wagner polarization, the total capacitance of a ceramic formed in an IBLC structure should be consistent with the total polarized charges or range of charge displacement under an external electric field. Therefore, limitation of the region for polarization due to a small insulating barrier might cause the *ε*′ values to decrease or remain constant. This result is similar to a cases of Li^+^, F^−^ codoped CCTO ceramics.^8^

**Fig. 8:**
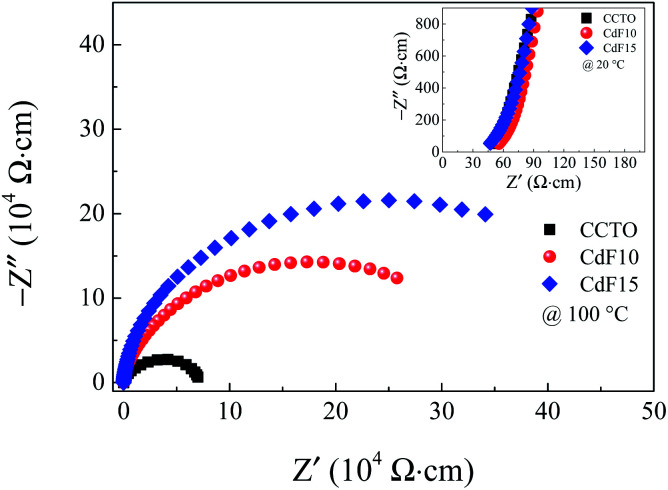
Impedance complex Z* plots at 100 °C of CCTO, CdF10, and CdF15 ceramics. Its inset shows a high-frequency Z* plots at 20 °C of these samples.

Impedance spectroscopy was performed to estimate the resistance of grains and GBs of CdF_2_ codoped CCTO ceramics to study the ceramic samples' electrical responses. An impedance complex (Z* = *Z*′ − *jZ*′′) plot can be made using the following relationship:1
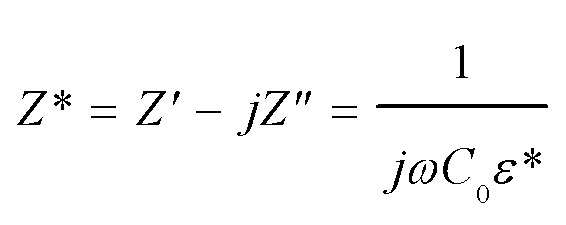
where *ω* = 2π*f* is the angular frequency. *C*_0_ = *ε*_0_*A*/*d* is the capacitance of free space. ε* is the complex dielectric permittivity consisting of a real part (*ε*′) or dielectric permittivity and an imaginary part (*ε*′′) or total loss factor. Resistances of grains (*R*_g_) and GBs (*R*_gb_) can be approximated from the nonzero intercept at high-frequency and a diameter of a semicircular arc of the Z* plot, respectively. However, for highly resistive ceramics, a semicircular arc is not formed at room temperature (RT). Thus, we considered *R*_gb_ at a relatively high temperature. The Z* plots at 100 °C for CCTO, CdF10, and CdF15 ceramics are given in Fig. 8. It is notable that the *R*_gb_ values of the doped ceramics are much larger than that of CCTO. Additionally, *R*_gb_ significantly increased with dopant concentration. The *R*_gb_ values of CCTO, CdF10, and CdF15 ceramics are listed in Table 2 as 5.52 × 10^4^, 2.86 × 10^5^, and 4.32 × 10^5^ Ω cm, respectively. As shown in the inset of Fig. 4(a), the reduction of tan *δ* at low-frequency might be caused by an enhanced *R*_gb_. This result closely agrees with previously published results, which suggest that the trend of *R*_gb_ and low-frequency tan *δ* is opposite.^4,6–11,29^ According to Mao *et al.*,^43^ a Cu-rich phase found at GB layers can reduce tan *δ* values to relatively low values, ∼0.048–0.083. This reveals that the enhanced GB response is due to a Cu-rich phase observed at GBs. Other researchers also reported a similar tendency.^6–8,27^

As previously mentioned, a Cd^2+^–F^−^ clustering (structure I of Fig. 6) is existed. This cluster might also suppress the charge migration, resulting in a reduction of DC conduction in the CdF_2_ codoped CCTO ceramics. Owing to lowering the DC conduction, the tan *δ* of these ceramics is dropped. Therefore, the enormously enhanced dielectric properties of CdF_2_ codoped CCTO ceramics can be primarily attributed to the decomposition of a Cu-rich phase and other related phases located at GB layers and the first blocking cluster inside the grains. As shown in the inset of Fig. 8, the *R*_g_ of the doped ceramics changed slightly compared to that of the CCTO ceramic. *R*_g_ values of CCTO, CdF10, and CdF15 ceramics were 50, 56, and 47 Ω cm, respectively. The presence of electrically heterogeneous regions between grains and GBs revealed by Z* plots supports the IBLC model as the primary origin of the colossal dielectric response in CCTO.^4,5,43^

**Fig. 9:**
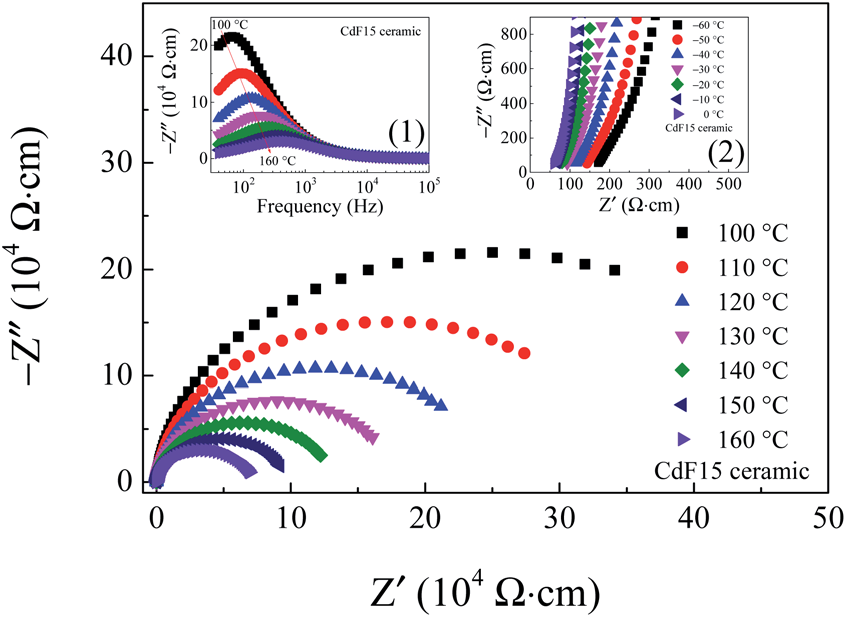
Impedance complex Z* plots of the CdF15 ceramic over a temperature range of 100–160 °C. Inset (1) shows the frequency dependence of *Z*′′ over this temperature range and inset (2) shows high-frequency Z* plots over a temperature range of −60 to 0 °C, respectively.

Variations of *R*_g_ and *R*_gb_ at several temperatures were examined. The results are shown in Fig. 9 and its insets. Over a relatively high-temperature range, the Z* spectra of the selected CdF15 sample consist of one symmetric semicircular arc, indicating the contribution of the grain boundary.

Clearly, the diameter of the semicircular arc of Z* plots is reduced by increasing the measuring temperature. This result indicates a significant decrease in *R*_gb_ with increased temperature. The decrease in *R*_gb_ can be determined using the frequency dependence of −*Z*′′ as well [inset (1)]. Simultaneously, by considering the high-frequency and relatively low-temperature, nonzero intercept indicates the electrical response inside the grains shift to a lower *Z*′-axis as soon as the temperature increases [the inset (2)]. This shifting spectrum shows that *R*_g_ variations have the same tendency as *R*_gb_ in a low-temperature range. This electrical response is usually observed in CCTO and related ceramics, indicating an IBLC structure.^4–8,10,11^

Temperature-dependent variations of *R*_g_ and *R*_gb_ can be used to estimate the conduction activation energies of grains (*E*_g_) and GBs (*E*_gb_) through a calculation using the Arrhenius law as follows:2
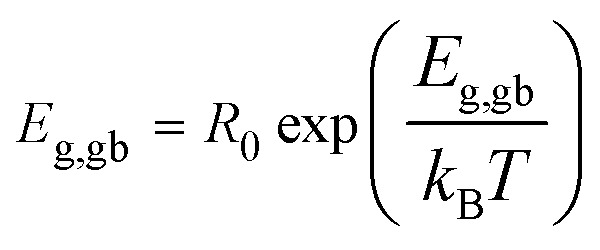


**Fig. 10:**
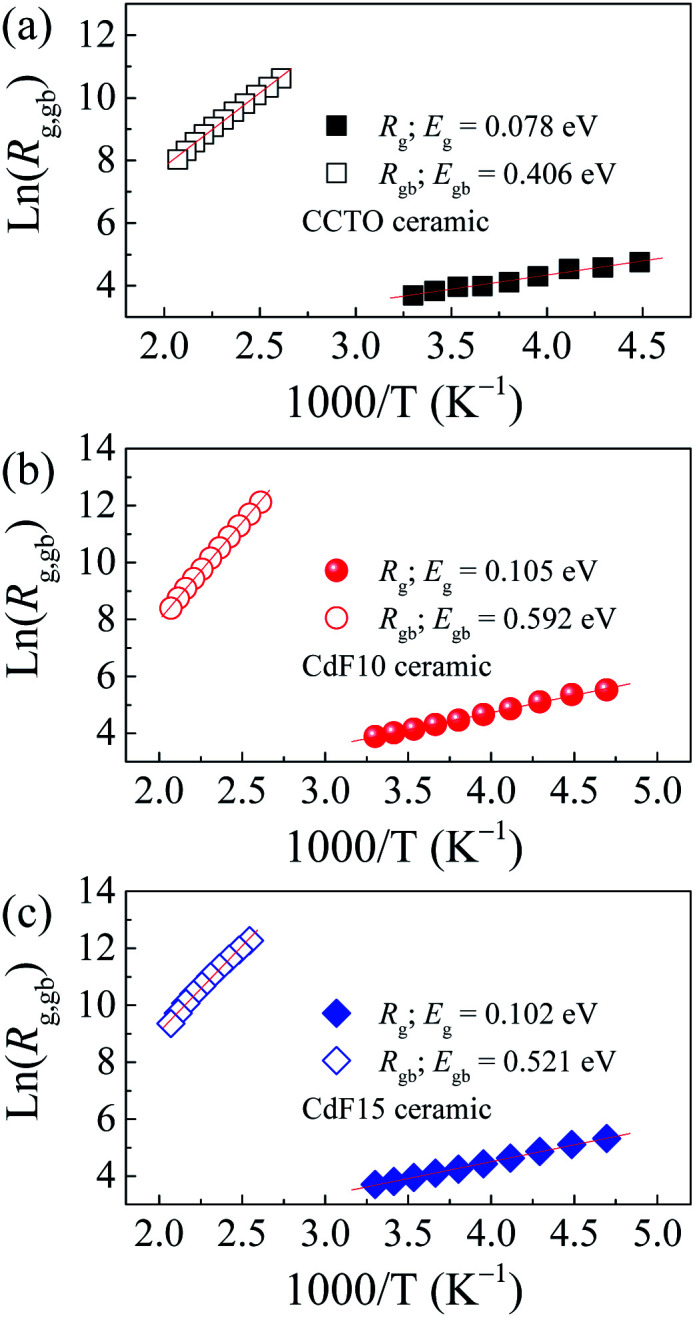
Arrhenius plots of *R*_g_ and *R*_gb_ values of (a) CCTO, (b) CdF10, and (c) CdF15 ceramics.

*R*_0_ is a pre-exponential term. Also, *k*_B_ and *T* are the Boltzmann constant and absolute temperature, respectively. Temperature dependencies of *R*_gb_ (open symbols) and *R*_g_ (solid symbols) for CCTO, CdF10, and CdF15 ceramics are illustrated in Fig. 10(a–c). This figure shows that the temperature dependencies of *R*_g_ and *R*_gb_ follow an Arrhenius relationship well. The slopes of fitted lines were used to estimate *E*_g_ and *E*_gb_. As shown in Table 2, the *E*_g_ of CCTO, CdF10, and CdF15 ceramics were 0.078, 0.105, and 0.102 eV, respectively. *E*_gb_ of these three samples were also considered. These values for the CCTO, CdF10, and CdF15 ceramics were 0.406, 0.592, and 0.521 eV, respectively.

The quite divergent values of *E*_g_ and *E*_gb_ show the dominance of the IBLC effect in CdF_2_ codoped CCTO ceramics.^43^ Interestingly, the *E*_gb_ of the doped samples increased compared to the CCTO ceramic. This result depicts an increase in the potential barrier height (*Φ*_B_) at GBs. Generally, a *Φ*_B_ located in the non-ohmic region possibly originates from either oxygen enrichment at the GBs and/or decomposition of metal oxide phases or precipitated phases during the sintering process.^27^

From the EDS, DFT, and impedance results, it is reasonable to propose that the enhanced *R*_gb_ observed in the CdF10 and CdF15 ceramics might be primarily caused by two significant factors. The first factor is precipitation of highly resistive insulating phases at GBs.^27,42,43^ A second factor arises from strong bonding between Cd^2+^ and F^−^ (structure I of Fig. 6) in the CCTO lattice, creating a small insulating cluster. These two factors have significant roles in blocking or inhibiting charge transfer inside the grains and across GB regions, resulting in reduced DC conduction due to long-range migration of charges. As a result, low-frequency tan *δ* might be suppressed to very small values.

## 4. Conclusions

The structural formations, dielectric properties and electrical responses of CdF_2_ codoped CCTO ceramics were systematically investigated. Crystal structure results indicate a single-pattern of the CCTO phase. CdF_2_ addition induces liquid-phase sintering that decomposes along grain boundary layers, resulting in a very great enlargement of grain sizes. DFT calculations indicate that a possible cause for liquid-phase sintering mechanisms might be the substitution of Cd^2+^ into Cu^2+^ sites, inducing an enhanced CuO–TiO_2_ eutectic liquid owing to excess Cu ions. The colossal dielectric properties of Ca_1−*x*_Cd_*x*_Cu_3_Ti_4_O_12−2*y*_F_2*y*_ ceramics were enhanced. Very-high *ε*′ ∼ 36 279–38 947 and low tan *δ* ∼ 0.024–0.033 values were accomplished in the doped ceramics. According to an impedance spectroscopy result, the largely reduced tan *δ* in the codoped CCTO ceramics could be induced by strongly increased grain boundary resistance and conduction activation energy of grain boundary. Therefore, improved dielectric properties in CdF_2_ codoped CCTO ceramics were created by enhancing grain boundary responses.

## Author contributions

J. B., P. T. and P. S. designed this project. J. B. and P. S. performed experiments and computations. J. B., J. P., J. M., S. K., P. T and P. S. analysed the computational and experimental data. J. B. and P. S. write the manuscript.

## Conflicts of interest

The authors declare no competing financial interests.

## Supplementary Material
